# Decoding Mitophagy in Breast Cancer: Biological Mechanisms, Dual Roles, and Clinical Implications

**DOI:** 10.3390/cells15181693

**Published:** 2026-09-18

**Authors:** Silvia Zunico, Giorgia Pisano, Cristina Della Bella, Maria Zeb, Luisa Cirillo, Martina Autiero, Raffaella Di Monda, Carmen Pacilio, Alessandra Leone, Michelino De Laurentiis, Stefania Cocco

**Affiliations:** 1 Department of Molecular Medicine and Medical Biotechnology, University of Naples Federico II, 80131 Naples, Italy; s.zunico@studenti.unina.it; 2 Department of Biology, University of Naples Federico II, 80125 Naples, Italy; giorg.pisano@studenti.unina.it; 3 Department of Breast and Thoracic Oncology, Division of Breast Medical Oncology, Istituto Nazionale Tumori IRCSS Fondazione G. Pascale, 80131 Naples, Italy; cristinadellabellaa@gmail.com (C.D.B.); luisa.cirillo@istitutotumori.na.it (L.C.); martina.autiero@istitutotumori.na.it (M.A.); raffaella.dimonda@istitutotumori.na.it (R.D.M.); c.pacilio@istitutotumori.na.it (C.P.); m.delaurentiis@istitutotumori.na.it (M.D.L.); 4 Clinical and Translational Oncology, Scuola Superiore Meridionale, 80138 Naples, Italy; m.zeb@ssmeridionale.it; 5 Experimental Pharmacology Unit, Istituto Nazionale Tumori IRCSS Fondazione G. Pascale, 80131 Naples, Italy; a.leone@istitutotumori.na.it

**Keywords:** mitophagy, breast cancer, triple-negative breast cancer, PINK1/PRKN pathway, BNIP3/NIX, mitochondrial quality control, reactive oxygen species (ROS), metabolic reprogramming, cancer stem cells (CsCs), therapy resistance, tumor microenvironment (TME), hypoxia, autophagy, OXPHOS

## Abstract

Mitophagy is a selective form of autophagy, essential to maintain cellular homeostasis by removing damaged and dysfunctional mitochondria. Several lines of evidence suggest that defective mitophagy is associated with various diseases, including breast cancer. Disruption of mitochondrial quality control contributes to the production of reactive oxygen species, causing DNA damage and consequently tumorigenesis. Moreover, mitophagy contributes to the regulation of other mechanisms implicated in tumor progression, such as metabolic adaptability, maintenance of stem-like properties, and metabolic remodeling of immune cells. In breast cancer, the role of mitophagy is context-dependent, with evidence suggesting it can both suppress or promote breast cancer depending on tumor stage, tumor microenvironment, or molecular profile. For this reason, while targeting mitophagy represents a promising therapeutic strategy in breast cancer, additional investigation is required to clarify its complex role and how mitophagy modulation can be effectively translated into treatment strategies. This review aims to report on the main evidence on the role of mitophagy in breast cancer, exploring mitophagy-targeted approaches in order to potentially overcome drug resistance in breast cancer.

## 1. Introduction

Breast cancer is a heterogeneous disease classified into distinct molecular subtypes based on receptor expression profiles, including luminal A (estrogen receptor [ER]+ and/or progesterone receptor [PR]+, Human Epidermal Growth Factor Receptor 2 [HER2]−, low Ki-67), luminal B (ER+ and/or PR+, HER2+/−, high Ki-67), HER2-enriched (ER−/low, PR−/low, HER2+), and triple-negative breast cancer (TNBC) (ER−, PR−, HER2−) [1]. Despite major advances in breast cancer treatment, including surgery, chemotherapy, radiotherapy, and targeted therapies, drug resistance still impairs effective breast cancer management, highlighting the need for new therapeutic approaches [2]. Increasing evidence suggests that the dysregulation of regulated cell death (RCD) promotes tumor progression, survival, and treatment resistance [3]. Moreover, in addition to apoptosis, several non-canonical RCD-related processes, such as autophagy-dependent pathways, are increasingly being investigated for their potential to overcome resistance in breast cancer [4,5]. Mitophagy, a specific form of selective autophagy, is particularly important for maintaining mitochondrial quality [6]. Indeed, mitophagy works by removing damaged or dysfunctional mitochondria through lysosomal degradation, thereby helping cells preserve energy balance and survive under stressful conditions such as hypoxia, nutrient deprivation, or deoxyribonucleic acid (DNA) damage [7].

While several papers have already described, in detail, molecular mechanisms of mitophagy, we focus more deeply on the dual role of mitophagy, pro-survival vs. suppression in breast cancer, and on challenges of translational approaches of mitophagy modulators in clinical practice.

## 2. Molecular Mechanisms of Mitophagy

At the molecular level, mitophagy is mediated by two major pathways: the first is a ubiquitin-dependent pathway involving PTEN-induced kinase 1 (PINK1) and PRKN (PRKN) [8,9]. The second is a receptor-mediated pathway involving proteins such as BCL2 Interacting Protein 3 (BNIP3), BNIP3-like (BNIP3L/NIX), FUN14 Domain Containing 1 (FUNDC1), and Prohibitin 2 (PHB2) that directly link mitochondria to the microtubule-associated protein 1A/1B-light chain 3 (LC3)/GABARAP family proteins [10,11,12].

In the ubiquitin-dependent pathway, the loss of the mitochondrial membrane potential (ΔΨm), typically resulting from mitochondrial damage, prevents PINK1 import into the inner mitochondrial membrane, thereby blocking its PARL-mediated cleavage and degradation and promoting its accumulation on the outer mitochondrial membrane (OMM). PINK1 becomes active and generates a phospho-ubiquitin signal that recruits autophagy receptors on impaired mitochondria. At the same time, PINK1 also phosphorylates PRKN at Ser65, promoting its recruitment and E3 ligase domain activation to build ubiquitin chains on multiple outer mitochondrial membrane substrates [13,14]. Ubiquitinated mitochondria are detected primarily by the autophagy receptors Optineurin (OPTN) and Calcium-Binding and Coiled-Coil Domain 2 (CALCOCO2/NDP52), which recruit early autophagy proteins at the mitochondrial surface [15,16]. This process can be enhanced by TANK-binding kinase 1 (TBK1), which phosphorylates autophagy adaptors such as OPTN, NDP52 (referred to above as CALCOCO2/NDP52), and Sequestosome-1 (SQSTM1/p62) and finally supports the assembly program during PINK1/PRKN mitophagy [17]. OPTN promotes mitophagy in a TBK1-dependent manner by binding ubiquitin chains and LC3, thus facilitating downstream recruitment of the autophagy machinery, including the Class III PI3K complex I [18,19]. Ubiquitin-independent pathways are increasingly recognized as an important component of mitophagy biology. In this kind of mechanism, outer-membrane receptors directly bind LC3/ATG8 proteins via LC3-Interacting Region (LIR) motifs, bypassing the ubiquitin coat [20]. These receptors, such as BNIP3 (BCL2 Interacting Protein 3), NIX (NIP3-like protein X)/BNIP3L, and FUNDC1 (FUN14 domain-containing 1), depend on their LIR motifs for their interaction with LC3/GABARAP proteins, thereby facilitating recruitment of the upstream autophagy machinery [9,12]. Recent evidence suggests that BNIP3/NIX not only function as LC3 receptors but also promote recruitment of upstream autophagy initiation machinery, including WIPI1 and ATG13, therefore facilitating phagophore formation [21]. BNIP3/NIX can also inhibit MTOR signaling to activate canonical mitophagy, while FUNDC1 exclusively recruits ULK1 [22]. Another example is Transforming Growth Factor Beta 1 (TGFβ1)-induced mitophagy, which requires Phospholipid Scramblase 3 (PLSCR3)-dependent cardiolipin externalization, working in synergy with BNIP3L/NIX, BNIP3, and FUNDC1-mediated mechanisms [23]. Another example is Prohibitin 2 (PHB2), which appears able to bind LC3 through the LIR domain upon mitochondrial depolarization [24].

Regardless of the specific molecular mechanism that triggers mitophagy, completion of the process requires proper autophagosome formation and endosomal sorting complex (ESCRT-III) machinery, particularly the CHMP2A and CHMP4B subunits, together with the AAA-ATPase VPS4, to mediate membrane abscission and phagophore sealing [25].

## 3. Dual Role of Mitophagy

Increasing evidence indicates that mitophagy is frequently altered in human cancers and can influence tumor development, either promoting or suppressing, depending on the stage of the disease and the surrounding cellular context [26]. In general, accumulating evidence suggests that the accumulation of intracellular reactive oxygen species (ROS) is associated with both tumor promotion and progression. In the early phases of tumorigenesis, mitophagy seems to act as a protective quality control program by clearing damaged mitochondria, thereby limiting the generation of ROS and preserving genomic stability [27]. However, in established or progressing tumors, mitophagy may support cancer cell survival by helping malignant cells to adapt to hostile conditions such as hypoxia, nutrient deprivation, and mitochondrial damage caused by therapy, ultimately preserving metabolism and stress tolerance. Overall, the role of mitophagy in cancer seems to depend on a delicate balance between the clearance of harmful or dysfunctional mitochondria and adaptive mechanisms that support survival programs and metabolic rewiring in stressed tumor cells [28].

### 3.1. Role of Mitophagy in Breast Cancer Suppression

Mitochondrial dysfunction is one of the main intracellular sources of ROS that are continuously produced by respiring mitochondria, and their level depends on the mode of electron transport in the respiratory chain [29]. Redox sensors in cytoplasmic and mitochondrial compartments are capable of dynamically and rapidly detecting changes in ROS levels, tuning the antioxidant defenses to respond to intra- or extracellular redox stressors that may perturb mitochondrial and cytosolic redox balance [30]. However, when the level of ROS is too high, the cell’s antioxidant defenses are overwhelmed. The accumulation of ROS causes oxidative damage to proteins, lipids, and DNA, promoting genomic instability. The effect is a vicious cycle in which oxidative stress further impairs mitochondrial function, amplifying ROS and supporting a tumor-promoting phenotype [31]. In this context, the collapse of the mitochondrial membrane potential, due to severe lipid peroxidation and protein damage, acts as a molecular distress signal that activates mitophagy as a tumor-suppressive quality-control mechanism by clearing damaged mitochondria [32]. In agreement with the cancer-suppressive role of mitophagy, defective mitophagy can drive ROS-linked inflammatory and metastatic phenotypes. In fact, excessive ROS production because of mitochondrial damage leads to severe modifications of proteins, DNA, and lipids, hence resulting in genomic instability and neoplastic transformation. So, mitophagy becomes relevant in many tumors, including breast cancer, as a quality control mechanism that eliminates dysfunctional mitochondria and limits ROS accumulation [10]. In particular, PRKN acts as a critical checkpoint in this defense pathway [33]. Loss-of-function studies have highlighted that PRKN deletion promotes genomic instability and increases proliferation and apoptosis resistance across several cancer models, including breast cancer [34,35]. The mechanism was first clarified by Tay et al., who found that restoring PRKN expression in PRKN-deficient breast cancer cell lines induced a G1 cell cycle block, without altering cyclin D1 or E levels, and, counterintuitively, increased CDK6 expression [36]. Consistent with a tumor-suppressive PRKN role, Gupta et al. showed that PRKN depletion, in preclinical breast cancer models, activates PI3K/AKT/mTOR signaling, lowering sensitivity to staurosporine-induced apoptosis and increasing vulnerability to PI3K/AKT inhibitors [37]. PRKN also seems to have a key role in the hypoxic response, since Liu et al. identified PRKN as an E3 ligase for HIF-1α, ubiquitinating it at lysine 477 and shortening its half-life. Accordingly, PRKN silencing stabilized HIF-1α and promoted metastasis, an effect reversible either by HIF-1α knockdown or by the small-molecule inhibitor YC-1 [38]. A further layer of regulation is epigenetic. Perego et al. found that the PRKN promoter is hypermethylated in breast cancer compared to relatively normal tissue, and its pharmacological demethylation with decitabine both restored PRKN expression and triggered an interferon/cGAS-STING-driven antitumor immune response [39,40]. Other findings showed that higher PRKN expression in breast tissues was correlated with chemosensitivity to antimicrotubule regimens and with improved disease-free and overall survival after neoadjuvant chemotherapy [41], reinforcing its candidacy as a prognostic marker.

The tumor suppressor *p53* is a transcriptional activator of PRKN; therefore, loss or mutation of *p53*, frequently observed in breast cancer, reduces PRKN expression, impairing mitophagy [42]. Zheng et al. demonstrated that the knock-out (KO) of *p53*, in hypoxia conditions, sensitized breast cancer cells to radiation by inhibiting PRKN-mediated mitophagy and blocking tumor cell survival [43].

Closely coupled with PRKN is its upstream regulator, PINK1, which acts as the primary sensor of mitochondrial damage. PINK1 and PRKN2 form a coordinated axis to maintain mitochondrial fidelity, and accumulating evidence suggests that PINK1 also exerts robust tumor-suppressive effects [44]. However, its role in cancer has contrasting views depending on the context, with increasing findings supporting a cancer promoter role in breast cancer [45].

Finally, the role of BNIP3-family receptors further illustrates how tightly mitophagy and ROS regulation are linked to breast cancer progression: in a mouse model of mammary tumorigenesis, BNIP3 loss caused mitophagy defects, increased mitochondrial ROS, and induced the activation of the HIF-1α–dependent pathway that promotes glycolytic and angiogenic programs that accelerated mammary tumor progression to metastasis [46].

Some studies suggest that TNBC progression may be linked to defective mitophagy. Specifically, in a TNBC mouse model, the reduced BNIP3 expression in tumors resulted in defective mitophagy with consequent increases in mitochondrial mass and ROS that activated HIF-1α and downstream target genes to promote tumor development. The *BNIP3* locus at chromosome 10q26.3 is also frequently deleted in metastatic progression of TNBC. Yet, *BNIP3* expression is retained in early-stage TNBC, and targeting of autophagy/mitophagy by small molecules such as chloroquine (CHQ) and isorhamnetin promotes apoptosis, suggesting that mitophagy is active in these cells during early stages of disease [47].

On the other hand, recent investigations have demonstrated elevated BNIP3 expression in breast cancer tissues compared with non-malignant breast samples [48]. However, the clinical relevance of BNIP3 expression in breast cancer remains unclear. Nuclear localization of BNIP3 in invasive breast carcinomas has been associated with smaller tumor size, lower histological grade, and estrogen receptor positivity. In contrast, cytoplasmic BNIP3 expression does not appear to correlate with established clinicopathological parameters in invasive disease [49]. Additionally, BNIP3-positive breast cancer patients show a reduced incidence of axillary lymph node metastasis, whereas increased BNIP3 expression in lymph node-negative cases has been linked to poorer clinical outcomes [48]. Overall, these findings highlight the complex and context-dependent role of BNIP3 in breast cancer and emphasize the remaining uncertainty regarding its biological and prognostic significance [50].

Other tumor suppressors implicated in breast cancer include *BRCA1,* which plays a critical role in maintaining mitochondrial integrity. Chen et al. reported that during mitochondrial stress, *BRCA1* is recruited to the mitochondrial outer membrane, where it plays an essential role in maintaining a healthy mitochondrial network. Consequently, *BRCA1* deficiency disrupts stress-induced mitophagy, increasing mitochondrial dysfunction and activation of the NLRP3 inflammasome, which creates a tumor-associated microenvironment, thereby facilitating tumor proliferation and metastasis [51].

Mitophagy failure has also been associated with bone metastasis: under hypoxia, MAPK1/3-driven Unc-51 Like Autophagy Activating Kinase 1 (ULK1) degradation attenuated mitophagy, leading to accumulation of ROS-generating mitochondria and NLRP3-mediated cytokine release. These changes can promote osteoclast differentiation and increased osteolytic bone metastasis [52].

Altogether, these studies highlighted the role of mitophagy as a tumor suppressor and the potential benefit of its induction to limit the consequences of mitochondrial dysfunction in cancer.

### 3.2. Pro-Survival Role of Mitophagy in Breast Cancer

Mitophagy can acquire a predominantly pro-survival role in established tumors by selectively eliminating dysfunctional and pro-apoptotic mitochondria. More broadly, advanced tumors often use mitophagy as a stress-adaptation program, enabling survival under chronic adversity. Consistent with this adaptive role, the activation of mitophagy can support long-term tumor cell persistence, treatment resistance, and disease relapse [53]. In hostile microenvironmental conditions, such as hypoxia and nutrient deprivation, mitophagy can act as a key mitochondrial quality-control pathway, limiting mitochondrial damage, preventing excessive ROS accumulation, thereby reducing the likelihood of necrosis and apoptosis [54]. Oxygen deprivation during hypoxia triggers HIF1α expression, which upregulates mitophagy via BNIP3 and NIX expression to counteract ROS production [55]. Ying Liu et al. also discovered that, in TNBC, under hypoxic conditions, HIF1α transcriptionally upregulates GPCPD1 that can translocate to the outer mitochondrial membrane, binding VDAC1, thereby increasing substrates for PRKN-mediated ubiquitination and enhancing mitophagy [56].

Other hypoxia-related mediators have been implicated in adaptive mitophagy activation. For instance, hypoxia-inducible mitochondrial ribosomal protein L52 (MRPL52), a nuclear-encoded mitochondrial protein that is transcriptionally upregulated by HIF1α, promotes PINK1/PRKN-dependent mitophagy to remove oxidatively damaged mitochondria, allowing cancer cells to achieve apoptotic resistance while triggering metastatic pathways [57].

A similar adaptive mechanism has been observed in nutrient deprivation or glucose starvation, in which the induction of a mitochondria-protective program, including PRKN-mediated mitophagy and fatty-acid oxidation, induces breast cancer survival and metastasis [42]. In this case, mitochondria and mitophagy assume a central role in cancer progression. The activation of the IGF-1/PI3K signaling pathway has been shown to increase BNIP3-mediated mitophagy, promoting the clearance of damaged mitochondria and preserving OXPHOS activity. Conversely, BNIP3 deficiency leads to mitochondrial dysfunction, elevated ROS, stabilization of HIF1α, and enhanced tumor growth in TNBC [46].

Moreover, during glucose deprivation, breast cancer cells can undergo a metabolic switch toward fatty acid oxidation to survive. This metabolic shift generates high oxidative stress, triggering a mesencephalic astrocyte-derived neurotrophic factor (MANF)-mediated mitophagy to clear damaged mitochondria and maintain cellular homeostasis [58].

The tumor microenvironment adds a further layer to this picture.

Under conditions of limited oxygen and nutrients, breast tumor cells impose oxidative stress on surrounding fibroblasts, leading to the downregulation of stromal Caveolin-1 (CAV1). This induces mitochondrial dysfunction, HIF-1/NFκB-dependent mitophagy/autophagy, and aerobic glycolysis within CAFs. As a result, cancer-associated fibroblasts (CAFs) undergo a metabolic switch, leading to the activation of mitophagy, autophagy, and aerobic glycolysis. Through this process, CAFs recycle biomolecules and produce metabolites, including lactate, pyruvate, and ketone bodies, supporting mitochondrial biogenesis and tumor growth [59]. This evidence shows that the TME is a complex ecosystem composed of cell populations of different entities that collectively shape tumor behavior [60]. In immune populations, mitophagy acts as a regulator of mitochondrial fitness and danger signaling: insufficient mitophagy allows mtROS and cytosolic mtDNA to accumulate, amplifying dysregulated inflammatory signaling and promoting functional exhaustion of CD8^+^ T cells and NK cells, whereas excessive mitophagy can remove mitochondrial danger signals and suppress cGAS–STING/NF κB activation [28]. In myeloid cells, mitophagy balances mtROS levels and inflammasome activity, which pushes tumor-associated macrophages (TAMs) toward an immunosuppressive M2-like state that suppresses the immune response. At the same time, when mitophagy goes awry in dendritic cells, it disrupts their metabolism and impairs their ability to cross-present antigens. This reduces T-cell activation and restrains immune cells from effectively entering the tumor [61].

Other mitophagy regulators have been reported to be implicated in breast cancer adaptation and aggressiveness.

Beyond its canonical mitochondrial function, PINK1 expression in breast cancer shows conflicting patterns across independent cohorts, which may itself be informative about its context-dependent role. Using paired tumor/adjacent-tissue qRT-PCR in 54 breast cancer specimens, Yaghoobi et al. reported a significant reduction in PINK1 mRNA in malignant tissues, an effect that also correlated with tumor mitotic rate [62]. On the other hand, Li et al. reached the opposite conclusion by evaluating PINK1 protein by tissue-microarray immunohistochemistry on 150 breast cancer tissues from different molecular subtypes. They showed that strong PINK1 staining correlates with higher histological grade. In addition, PINK1 mRNA is likewise higher in breast cancer cell lines than in the non-tumorigenic MCF-10A line. PINK1 knockout in the TNBC line MDA-MB-231 also reduced both proliferation and glycolytic capacity on Seahorse extracellular flux analysis, consistent with an oncogenic, glycolysis-supporting role for PINK1 in this specific subtype [63]. Reconciling transcript-level, protein-level, and functional data therefore remains an open problem for the field, since mRNA abundance, protein stability, and subcellular localization may each carry distinct biological information. On this last point, Berthier et al. showed that PINK1 subcellular distribution itself differs between normal and malignant breast tissue, since cancer samples displayed diffuse cytoplasmic staining with pronounced membrane localization, compared with a punctate cytoplasmic pattern and minimal membrane staining in normal tissue, suggesting that localization, rather than absolute expression level, may hold the more robust biomarker potential [64]. Reported discrepancies in PINK1 expression may reflect differences between bulk mRNA and protein-based detection, assessment of full-length versus cleaved PINK1, subcellular localization, mitophagic flux, tumor-cell composition, and subtype-specific metabolic program. Future studies should validate PINK1 and other mitophagy-related markers in well-annotated clinical breast cancer cohorts, integrating transcriptomic and protein-level assays, molecular subtype, stage, metastatic status, and treatment exposure. A comparable oncogenic signature has been described for FUNDC1. Immunohistochemistry showed strong cytoplasmic and nuclear-membrane FUNDC1 staining in breast cancer tissue, essentially absent from normal breast, and Kaplan–Meier analysis linked high FUNDC1 expression to significantly worse patient survival. Gain- and loss-of-function experiments confirmed a causal role, with FUNDC1 overexpression enhancing proliferation, migration, and invasion in vitro and its knockdown producing the opposite effect. Mechanistically, elevated FUNDC1 increased cytosolic Ca2+ influx from both the endoplasmic reticulum and the extracellular space, driving nuclear translocation of the transcription factor NFATC1, which directly activates BMI1. In confirmation of this effect, tumors co-expressing FUNDC1 and BMI1 carried a worse prognosis than those expressing either marker alone [65]. Notably, this Ca2+-NFATC1-BMI1 pathway operates independently of FUNDC1’s canonical mitophagy-receptor function, illustrating how a mitophagy protein can drive tumor progression through an entirely non-mitophagic mechanism.

Other findings indicate that FUNDC1 promotes mitophagy through ERK1/2 signaling, thus supporting tumor cell survival [66]. Collectively, these observations support a model in which mitophagy acts as a pro-survival, adaptive program in breast cancer, helping cells to better tolerate mitochondrial dysfunction and oxidative stress, while promoting proliferation, invasiveness and resistance to therapy [67]. In agreement with this hypothesis, it has been reported that inhibition of mitophagy in mitophagy-dependent breast cancer models can contribute to reducing aggressiveness [68]. Inhibition of Mucin-1 (MUC1), a protein localized to the outer mitochondrial membrane able to promote mitophagy, suppresses breast cancer cell proliferation in vitro and in vivo models, suggesting that some breast cancers could be susceptible to therapeutics targeting this pathway [69].

Finally, other evidence suggests that extracellular vesicles, such as exosomes, can transfer autophagy and mitophagy components to other cells, enhancing the aggressive behavior of cancer cells [70].

Moreover, stromal cells, including mesenchymal stem cells and immune cells, seem to transfer functional mitochondria to cancer cells via nanotubules or extracellular vesicles, sustaining OXPHOS and tumor growth in multiple cancer types, including breast cancer, highlighting a conserved mechanism by which tumor cells maintain energy production and metabolic plasticity [71,72].

**Figure 1:**
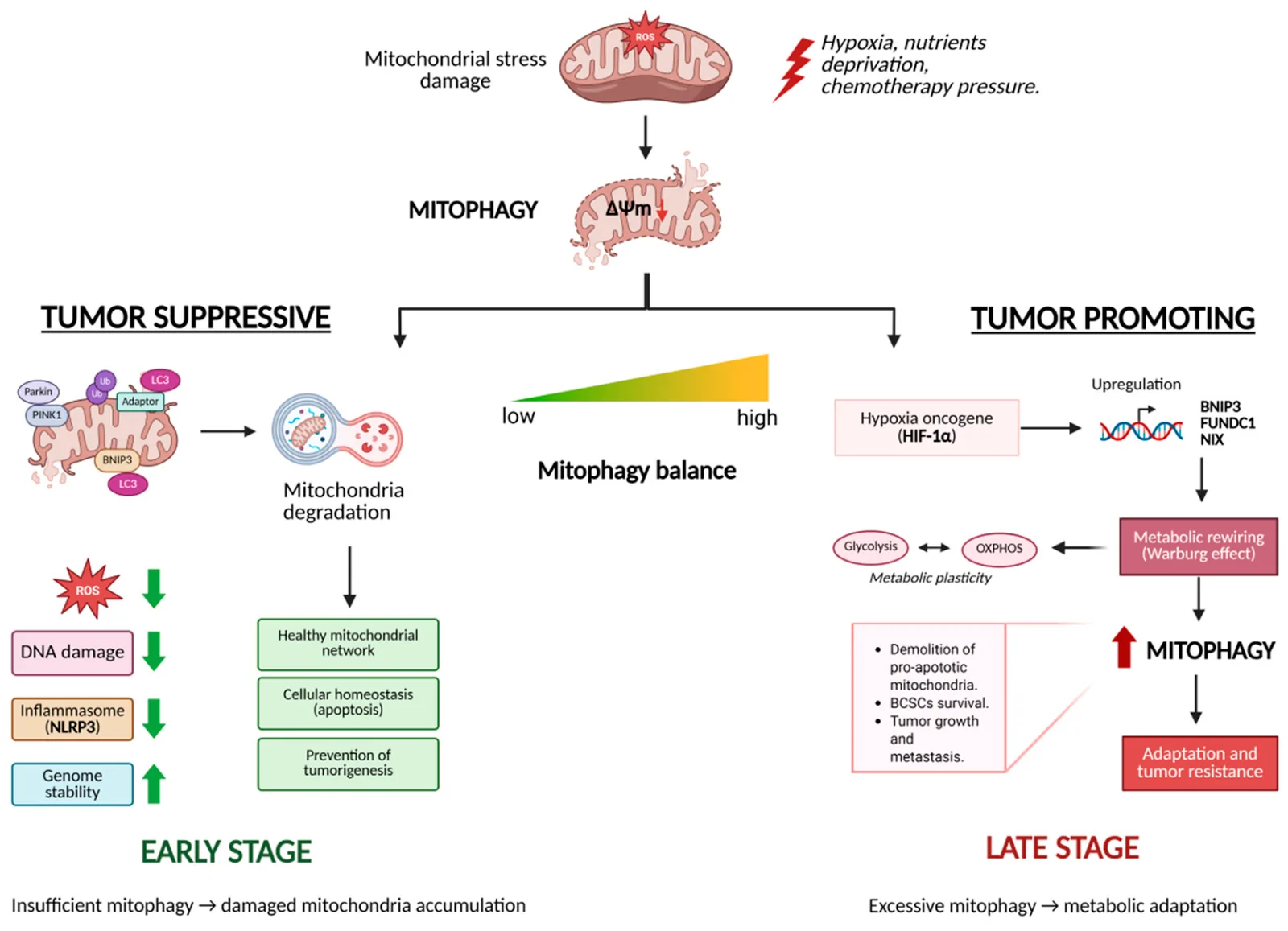
The dual role of mitophagy in tumor suppression and tumor progression.

The main mechanisms and role of mitophagy in both breast cancer suppression and survival are reported in Figure 1.

Mitochondrial stress induced by hypoxia, nutrient deprivation, or chemotherapy leads to mitochondrial damage and activation of mitophagy. The balance of mitophagy determines its functional outcome in cancer.

### 3.3. Determinants of the Tumor-Suppressive vs. Tumor-Promoting Switch

Section 3.1 and Section 3.2 described how mitophagy can shift toward either a tumor-suppressive or a tumor-promoting role in breast cancer. This dual behavior is not an intrinsic property of the pathway itself; it emerges from four interacting contextual variables. The first is disease stage and stress severity. In early stages, mitophagy mainly clears out damaged mitochondria, limits ROS accumulation, and preserves genomic stability, which supports tumor suppression [26]. As tumors become established, though, the same machinery gets redirected toward pro-survival metabolic adaptation, helping cells resist apoptosis and keep proliferating under hostile microenvironmental conditions [50].

Second, the type of stress determines which mitophagy receptor program gets engaged. PINK1/PRKN responds mainly to mitochondrial damage and depolarization, while BNIP3/NIX, upregulated via HIF-1α, mediates hypoxia-driven mitophagy. In breast cancer, losing BNIP3-dependent mitophagy under normoxia leads to superoxide accumulation, HIF stabilization, and metastatic progression; under hypoxia, by contrast, inducing it supports metabolic flexibility and invasion instead [49].

Third, treatment exposure shapes the outcome independently of tumor stage or stress type. Chemotherapy can damage mitochondria enough to trigger PINK1/PRKN-dependent mitophagy as a protective response, clearing pro-apoptotic mitochondria before cell death, a mechanism linked to chemoresistance in breast cancer models. In TNBC, however, PINK1-mediated mitophagy can instead promote PD-L1 degradation, which paradoxically increases sensitivity to immunotherapy [73].

Finally, molecular subtype seems to matter less on its own, and more as a proxy for underlying differences in hypoxia burden, metabolic demand, and treatment history across tumors. This may be why mitophagy-associated markers carry prognostic value in some subtypes, such as TNBC, but not others [67].

## 4. Mitophagy Contribution to Stem-like Cells (BCSCs)

Breast cancer stem cells (BCSCs) represent a sub-population characterized by enhanced tumor-initiating capacity, self-renewal, differentiation plasticity, and resistance to therapy [74]. Accumulating evidence has shown how increased mitochondrial turnover (including mitophagy) is a key component of BCSC state regulation and therapeutic resilience. Mitophagy seems to support the “stemness” of BCSC by removing damaged mitochondria, reducing ROS and preserving bioenergetic competence [75]. Consistent with a direct link between mitophagy and BCSC features, BNIP3/BNIP3L-high breast cancer cells exhibit higher basal mitophagy together with increased mammosphere-forming capacity, stemness-associated markers, metabolic activity, migration, and drug resistance, suggesting that elevated baseline mitophagy can enrich BCSC-like traits [76]. In addition, in therapy-pressure contexts, PINK1-mediated mitophagy seems to work as a protective drug-tolerant persistent (DTP) mechanism, increasing BCSC survival during/after treatment [77]. In this context, perturbation of mitophagy represents a valid pragmatic strategy to eradicate CSCs in breast cancer. Consistent with this approach, a mitochondria-interfering nanocomplex has recently been developed to downregulate PD-L1 expression, thereby boosting antitumor immunity while simultaneously impairing CSC survival under hypoxic conditions via AMPK-mediated pathways [78]. In addition, silencing ARIH1 (a mitophagy-associated E3 ligase) significantly attenuates cancer stem cell properties and tumor formation in breast cancer cells, while its overexpression promotes EMT and invasion [79]. At the same time, ARIH1 overexpression in BC enhances cytotoxic T-cell infiltration and improves the efficacy of PD-L1 blockade, while its loss has been linked to immune checkpoint blockade resistance in TNBC models [80].

**Figure 2:**
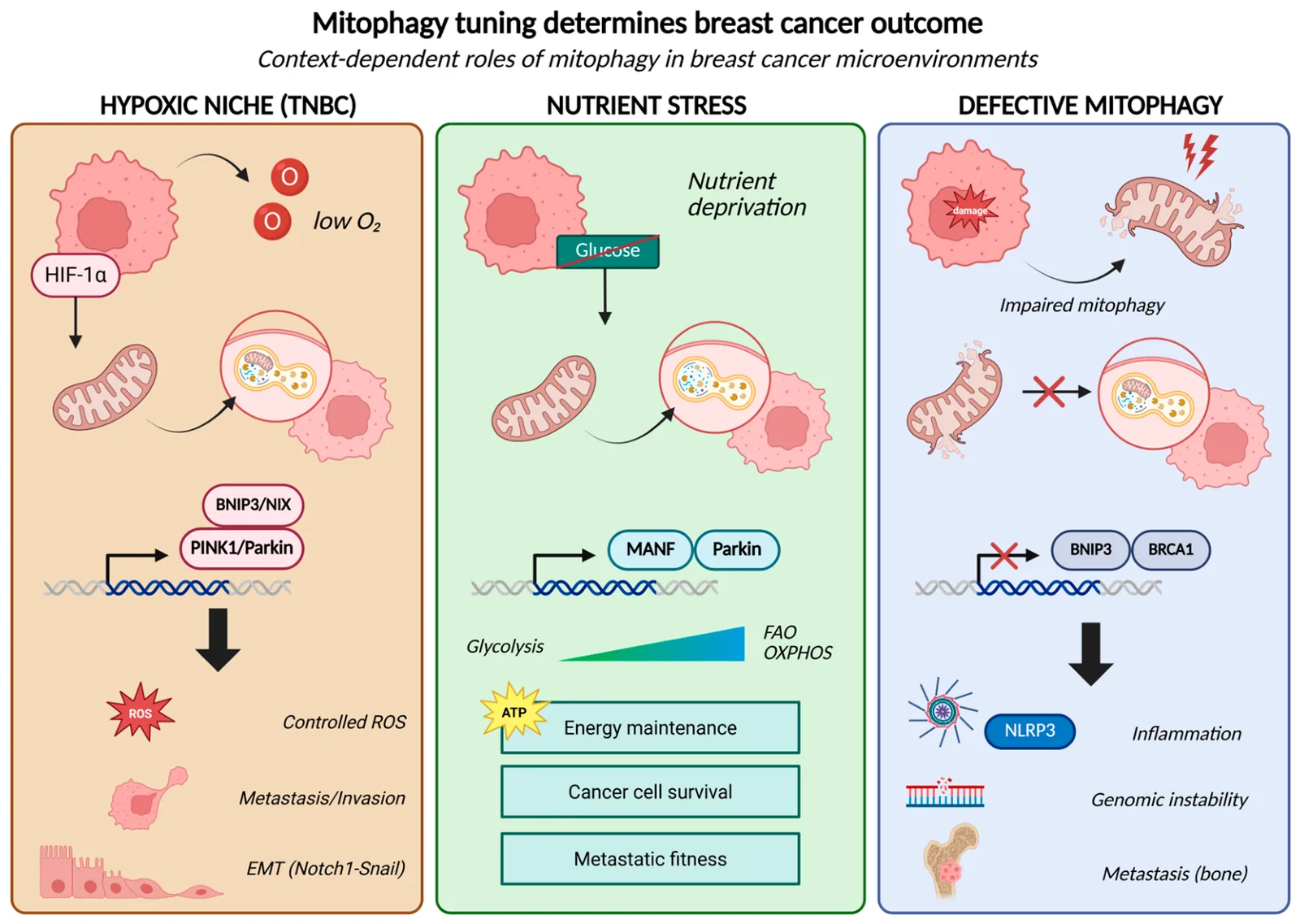
Context-dependent roles of mitophagy in the breast cancer microenvironment.

In Figure 2 context-dependent roles of mitophagy in the breast cancer microenvironment are reported.

Mitophagy leads to distinct outcomes depending on the cellular context. In hypoxic niches, particularly in TNBC, HIF-1α activation promotes mitophagy through BNIP3/NIX and PINK1/PRKN pathways, contributing to controlled ROS production and supporting cancer progression, including EMT. Under nutrient deprivation, mitophagy supports metabolic adaptation by maintaining energy homeostasis through a shift between glycolysis and oxidative phosphorylation. Conversely, defective mitophagy leads to the accumulation of impaired mitochondria, resulting in increased inflammation (via NLRP3 activation), genomic instability, and enhanced metastatic potential.

## 5. Mitophagy in Therapeutic Resistance and Stress Adaptation

Several evidence suggests that exposure to therapeutic agents can trigger adaptive autophagy/mitophagy mechanisms that, in the long term, can facilitate the development of therapy resistance [81]. Since persistent chemotherapy usually damages mitochondria, in response to this, many cancer cells may induce mitophagy to remove impaired organelles, thereby avoiding apoptosis. Consequently, blocking this protective process could sensitize breast cancer cells to treatment [82]. Some preclinical studies support this approach, since it has been shown that liensinine, a potent inhibitor of late-stage autophagy and mitophagy, by preventing autophagosome–lysosome fusion, enhanced doxorubicin-induced apoptosis via DNM1L-mediated mitochondrial fission, and suppressed xenograft tumor growth [83]. A similar strategy was adopted by Naso et al., who demonstrated that PINK1/PRKN-dependent mitophagy is induced by doxorubicin treatment in different breast cancers and that inhibiting this mitophagy, via miR-218-5p targeting PRKN, increases the cells’ sensitivity to doxorubicin [84]. Pu et al. showed that the antipsychotic medication chlorpromazine showed some tumor-growth inhibition in TNBC models by triggering incomplete autophagy and PINK1/PRKN-mediated mitophagy. At the same time, inhibition of autophagy/mitophagy augmented chlorpromazine anticancer effects, indicating these processes may have cell-protective roles [85]. In this context, it has been shown that bevacizumab-induced hypoxia activates HIF-1α-dependent metabolic reprogramming, including PDK1 upregulation, which in turn promotes mitophagy and supports tumor cell adaptation to therapeutic stress [86].

Mitophagy also determines the ability of the tumor to elude the immune system. In particular, PD-L1 protein can be directed to the mitochondria and degraded via PINK1-dependent mitophagy by the ATPase family AAA domain containing a 3A (ATAD3A) factor that halts this degradation. In agreement with this hypothesis, Xie et al. showed that modulation of the ATAD3A-PINK1 axis can force PD-L1 elimination, representing a significant opportunity to sensitize “cold” tumors to immunotherapy [73].

The study made by Zhang et al. previously reported reinforces the broader idea that interfering with mitochondrial fitness is a genuinely viable way to strip PD-L1 off the tumor surface. The authors’ mitochondria-targeting nanocomplexes (CuET/ICG) interfere with mitochondrial energy metabolism, triggering AMPK activation and downregulating membrane PD-L1 in both tumor cells and cancer stem cells under hypoxic conditions, in a mouse breast cancer model combined with photodynamic therapy [78].

## 6. Mitophagy Across Breast Cancer Histotypes

Mitophagy programs are not histotype-specific; however, breast cancer histotypes may differ according to the selected mitophagy route under the dominant stress (hypoxia, nutrient deprivation, or chemotherapy pressure) and whether damaged mitochondria are successfully eliminated (high mitophagic flux) or whether they accumulate after an impaired process (ROS/inflammation). Apparently, this explains the dual role of mitophagy in breast cancer as both a tumor suppressor and promoter, reflecting the different engagement of PINK1-PRKN (import stress/damage-linked) and BNIP3/NIX (hypoxia-linked receptor) programs, as stress can drive the switch between the two modules rather than a mere on/off behavior [67].

In luminal (ER+) and HER2-enriched breast cancers, there is less evidence related to a tumor-suppressive or pro-survival role of mitophagy. Because of this, it is more useful to think about mitophagy in terms of cellular state and stress, rather than strict subtype differences. While the core mitophagy machinery is shared across breast cancer subtypes, the selective pressures that shape its activity, such as baseline hypoxia, nutrient availability, and treatment exposure, likely vary between subtypes [87]. As a result, mitophagy-related signals observed in patient samples may reflect tumor context or stage more than intrinsic subtype identity. Supporting this idea, the study of Lefort et al. found that the autophagy marker LC3B seems to have a prognostic value specifically in TNBC, but not in luminal or HER2-enriched tumors [88]. This evidence suggests that mitochondrial and autophagy dependencies might be more strongly selected in certain TNBC histotypes than in other breast cancer subtypes. In conclusion, clear subtype-specific differences in mitophagy pathways in luminal and HER2-enriched cancers are still not well defined; therefore, more studies, especially those that measure mitophagy’s dynamics and are stratified by subtype, are strongly needed. Until then, mechanistic conclusions should rely on flux-aware assays rather than static marker measurements.

## 7. Mitophagy Biomarkers in Breast Cancer

Mitophagy-related markers can be explored as prognostic and, in some cases, predictive biomarkers, but their clinical usefulness is still too far to be integrated into clinical practice. A key distinction is between static measures of autophagy/mitophagy marker expression and assays that directly capture the mitophagic flux [89]. Human clinical cohort analyses reveal that mitophagy effectors vary dynamically across the breast cancer spectrum rather than operating as simple binary switches [90]. Epigenetic regulation plays a major role: PRKN promoter hypermethylation is frequently observed in breast cancer tissues, whereas its demethylation restores expression and triggers antitumor immune responses. Higher PRKN levels further correlate with increased chemosensitivity and improved survival following neoadjuvant chemotherapy [40].

In patient samples, immunohistochemical (IHC) scoring of LC3B puncta in residual tumors after neoadjuvant chemotherapy has been linked to relapse-free and overall survival. However, LC3B factors mainly reflect autophagosome abundance or redistribution, rather than selective removal of mitochondria. Combining LC3B puncta with nuclear HMGB1 has been shown to improve prognostic stratification in large adjuvant chemotherapy cohorts, but this approach still represents an indirect surrogate of mitophagy. In addition, both markers are influenced by immune infiltration, which can complicate interpretation of any “mitophagy score” [91].

Some individual regulators of mitophagy also show prognostic associations. For example, as reported above, MANF has been reported to enhance PRKN activity under glucose starvation, supporting mitophagy and fatty-acid oxidation; however, increased MANF expression is linked to poorer outcomes [58]. Conversely, loss of BNIP3 promotes tumor progression and metastasis in breast cancer models and may have stratification value in human TNBC. This aligns with broader studies that identify BNIP3 (alongside proteins like PRKN) as part of mitophagy-related biomarker panels in breast cancer tissues [48]. However, their clinical relevance seems context- and localization-dependent. In invasive breast cancer, loss of BNIP3 was associated with a higher frequency of lymph node metastases and increased proliferative rate [49]. Conversely, in a separate transcriptomic study, elevated BNIP3 expression was associated with poorer outcome [92].

Other potential biomarkers are FUNDC1 and PINK1. In a cohort of 102 breast cancer patients, FUNDC1 immunoreactivity was detected predominantly in the cytoplasm and cell membrane of tumor tissues, whereas normal breast tissues showed absent or weak staining. Higher FUNDC1 expression was associated with larger tumor size and advanced stage [65].

Regarding PINK1, a retrospective analysis of TCGA-BRCA tumor tissues (*n* = 1070) found that higher tumor PINK1 expression was associated with worse overall survival [63]. Recent integrative transcriptomic and spatial analyses in triple-negative breast cancer identified PINK1 within a prognostic gene signature. In an immunohistochemistry-validated retrospective cohort of 48 patients, high tumor-cell PINK1 expression was associated with recurrence and shorter 5-year disease-free survival, including after adjustment for stage and nodal status [93]. For instance, a transcriptomic approach identified “mitophagy gene signatures” to stratify patient risk and identify drug-relevant phenotypes, including models derived from single-cell data in TNBC, as well as broader breast cancer prognostic cohorts [94]. Moreover, a further study integrating multi-omics, single-cell, and spatial transcriptomic analyses demonstrated a link between mitochondrial inner-membrane protein (IMMT) expression and mitophagy-related signatures, immune-microenvironment features, mitochondrial function, clinical prognosis, and drug sensitivity [95]. Collectively, the above mentioned studies highlight that only the integration of multi-omics assays, including epigenetic, genomic, and proteomic/metabolic techniques, can capture the full regulatory complexity of context-dependent mitophagy, thereby enabling the identification of clinically actionable biomarkers [96].

However, these signatures often capture a mixture of cellular states, such as stress responses, immune activity, and proliferation, rather than directly measuring mitochondrial turnover. For this reason, it is important to avoid overinterpreting expression-based data as evidence of “high” or “low” mitophagy employing functional assessments of mitophagic flux that require dedicated tools, such as mt-Keima or mito-QC reporters, which can directly track mitochondrial delivery to lysosomes [97,98]. In this case, careful experimental design is essential because results depend on the reporter system used, including differences in sensitivity to PINK1/PRKN-mediated pathways, and robust conclusions typically require lysosomal inhibition controls and complementary quantitative approaches, such as flow-based assays [99]. These limitations should be clearly acknowledged when interpreting mitophagy in clinical or translational studies.

## 8. How to Measure Mitophagy in Breast Cancer Models: Why the Common Assays Mislead

Failure to measure mitophagy flux is a major confounder in mitophagy studies and likely explains much of the apparent “dual role”, tumor-suppressive vs. tumor-supportive, because many commonly used readouts capture mitophagy induction signals, or general autophagy, rather than mitophagy flux. Many widely used readouts in breast cancer models are static and cannot discriminate increased autophagosome formation from late-stage blockade without lysosomal inhibition controls, which is a major source of apparent “dual roles” across studies. In practice, static markers such as LC3 puncta, LC3-mitochondria colocalization, p62/SQSTM1 levels, PRKN expression/translocation, TOM20 loss, or “mitophagy gene signatures” can be misleading without lysosomal flux controls because they cannot distinguish increased autophagosome formation from impaired autophagosome–lysosome fusion, nor decreased mitochondrial mass from altered biogenesis/fission–fusion. Methodological pitfalls and probe/colocalization limitations are well described in foundational microscopy-focused guidance [89,100].

In addition, relying solely on PINK1 and PRKN (expression, total protein levels, or mitochondrial recruitment) to interpret “mitophagy activation” is risky because they can also pick up mitophagy-independent MQC routes, such as MDVs, or proteasome-driven turnover of outer mitochondrial membrane proteins (OMMAD), not to mention downstream blocks [101].

Similarly, nutrient-stress survival programs, attributed to mitophagy, require flux-aware assays to support a causal pro-survival mitophagy mechanism rather than generic autophagy induction. For functional quantification, the most widely adopted mitophagy flux reporters are pH-sensitive mitochondrial Keima systems (mt-Keima/mKeima), which directly report mitolysosome formation by ratiometric excitation shifts and have been validated in vivo and in vitro, and the tandem-fluorescent mito-QC reporter (mitochondria-targeted mCherry-GFP) that reports lysosomal delivery by GFP quenching with retained mCherry signal [102]. Importantly, direct comparisons show mt-Keima can detect PINK1–PRKN mitophagy with higher sensitivity than mito-QC under some conditions, offering a plausible technical basis for conflicting conclusions in the literature [102].

Beyond imaging, quantitative flow-based flux methods can strengthen breast cancer studies by enabling population-level measurements and multiplexing with viability/ROS: a MitoTracker Deep Red + lysosomal inhibitor approach provides a practical mitophagy-flux readout, and newer flow protocols using the mito-QC reporter further increase throughput and robustness for mitophagy quantification and drug testing [103]. Finally, because some mitochondrial quality control in cancer may occur via non-lysosomal/secretory routes, such as extracellular vesicle-associated export of mitochondrial components, assays limited to lysosomal reporters alone may undercount total mitochondrial clearance, further complicating interpretation of “high vs. low mitophagy” across breast cancer contexts [104].

Systematic clinical cohort analyses that compare key mitochondrial autophagy molecules across subtypes, disease stages, and detection levels (transcript vs. protein vs. subcellular localization) are essential to resolve the inconsistencies in the current literature. To date, no prospective clinical trial in breast cancer has directly measured mitophagic flux (e.g., via mt-Keima or functional assays) to link real-time dynamic clearance with therapeutic response. Static biomarkers like PINK1, PRKN, BNIP3, or LC3 cannot be interpreted on their own as definitive proof of active mitophagy. Future translational work will therefore need to pair multi-omic clinical profiling with flux-aware methodologies to build validated, subtype-specific prognostic and predictive algorithms.

Table 1 summarizes clinical and translational studies evaluating mitophagy-related markers in breast cancer.

## 9. Discussion

In recent years, several studies have substantiated the role of autophagy in cancer progression, including breast cancer. However, the implications of selective autophagy mechanisms, such as mitophagy, continue to be underestimated, compared to neurodegenerative and cardiological diseases. In part, this effect is due to the challenges in measurement assays of mitophagic flux.

Rather than viewing the complexity of mitophagy as a barrier, the evidence collected until now suggests that the context-dependent role of mitophagy in breast cancer allows for the exploitation of these pathways by applying a stratified approach: inhibiting mitophagy in stress-adapted, advanced tumors to sensitize them to standard therapies, or conversely, inducing/restoring mitophagy to clear damaged mitochondria, limiting ROS-driven metastasis.

In advanced/aggressive tumors, in particular TNBC, blocking mitophagy could represent a highly effective way to chemo-sensitize breast cancer cells. At the moment, the available strategies include hydroxychloroquine (HCQ)-derivatives or liensinine [83], allowing the targeting of autophagic flux to combine with standard therapeutic agents. In truth, several preclinical studies have already shown the efficacy of these combinations, measuring their effect mainly in terms of autophagy, rather than mitophagy [105]. For instance, HCQ is currently being studied in preclinical and clinical trials to potentially sensitize tumors to chemotherapy because of its ability to induce apoptosis and prevent their survival under stress conditions by inhibiting late-stage autophagosome–lysosome fusion, specifically in autophagy [106]. Meanwhile, encouraging results were derived from the Phase II CLEVER trial (NCT03032406) in which a combination of HCQ and Everolimus eliminated ~80–100% of dormant breast cancer cells in bone marrow, significantly reducing metastatic recurrence by blocking autophagy-mediated survival [107]. Moreover, the ongoing Phase II PALAVY trial (NCT04841148) is evaluating the role of autophagy inhibition by HCQ, along with Palbociclib and Avelumab, in order to eliminate dormant disseminated tumor cells (DTCs) after surgery in breast cancer patients. In parallel, the NCT03774472 phase I trial, testing HCQ at 400, 600, and 800 mg/day combined with continuous palbociclib and letrozole in 14 evaluable ER+/HER2-metastatic breast cancer patients, using a 3 + 3 dose-escalation design, was designed on the basis that low doses of palbociclib activate autophagy and reverse the initial G1 cell-cycle arrest, while HCQ converts this into on-target senescence instead [108]. However, the translational implications of these strategies are still far from being implemented in clinical practice.

Targeting the metabolic switch during hypoxia, through the pharmacological inhibition of regulators such as BNIP3/NIX and GPCPD1 or HIF-1α and PDK1, could be an effective approach to shutting down this adaptive mitophagic shield. However, recent research approaches are more focused on targeting metabolism by modulation of the HIF1a pathway. For instance, HIF-1a inhibitors, such as Digoxin, have been evaluated in clinical trials for their ability to modulate the hypoxic microenvironment and relative transcriptomic signatures [109]. Also, a benzenesulfonamide derivative, acting as a downstream target of HIF-1a, has been tested in advanced solid tumors, including TNBC [110]. Other agents able to inhibit the monocarboxylate transporter (MCT) 1-mediated lactate transport have shown cytostatic/cytotoxic effects in preclinical models and are under clinical evaluation, with promising results [111].

Another potential successful approach includes the targeting of mitophagy or autophagy in tumors overexpressing the PI3K/AKT/mTOR pathway. The hyperactivation of the PI3K/Akt/mTOR pathway is one of the most frequent oncogenic alterations in breast cancer, particularly in Luminal B, HER2-enriched, and TNBC subtypes. When the PI3K/Akt/mTOR pathway is hyperactive, tumor cells run their metabolism in overdrive, generating toxic ROS as a byproduct that would normally be lethal [112]. In this context, it is plausible that tumor cells may activate mitophagy as a means of limiting ROS production, suggesting that mitophagy inhibition could represent an effective therapeutic strategy in PI3K/Akt/mTOR hyperactivated tumors.

Conversely, the above-mentioned clever trial is based on the idea that mTOR inhibition is one of the most potent inducers of autophagy, and relative activation of autophagy may itself be a key resistance mechanism to mTOR inhibition with everolimus. In agreement, supporting preclinical data showed that inhibition of the PI3K/Akt/mTOR pathway induces strong autophagy activation; therefore, its inhibition by HCQ enhances cell death and tumor regression [106]. This is built on a broader research effort with multiple HCQ trials, including five phase I/II trials in different oncological diseases.

As extensively mentioned before, one of the most promising translational applications of mitophagy modulation lies in the eradication of BCSCs. Despite research into mitophagy’s role across various stem cell developmental contexts, the precise role and mechanism of mitophagy specifically in maintaining stemness and regulating differentiation in BCSCs remain to be fully elucidated. In other words, most of the current evidence is correlative/associative (elevated mitochondrial machinery in stem-like cells) or built on adjacent mitochondrial dynamics proteins (Mitofusin, FIS1) rather than on the core mitophagy receptors (BNIP3, NIX, FUNDC1, PINK1/PRKN).

While inhibiting mitophagy is ideal for advanced, stress-adapted tumor masses, inducing mitophagy represents a sophisticated alternative in specific molecular configurations.

As previously described, the role of mitophagy is context-dependent, and mostly in early tumors, the ROS rescue role of mitophagy could be potentiated in order to prevent the switch to advanced stages. However, mitophagy inducers have not yet been studied for oncological approaches, but several compounds evaluated in other diseases could be potentially repurposed in cancer.

For instance, Urolithin A, a postbiotic supplement typically used to improve muscle strength and immune function, has been reported to increase phospho-PRKN, mitophagy, and mitochondrial biogenesis genes [113]. More recently, the MitoIMMUNE clinical trial presented at ASCO 2024 showed that urolithin A shifts circulating immune cells toward a less exhausted, less inflammatory phenotype, expanding naive-like and stem-like CD8+ T cells with improved fatty acid oxidation capacity. The authors explicitly propose this as a rationale for combining urolithin A with cancer immunotherapy, on the premise that healthier T-cell mitochondria support better antitumor immune function [114]. In parallel, Spermidine, a natural dietary supplement used as a cardioprotector, has been shown to extend lifespan and preserve cardiac function in aged and hypertensive rodent models, with the protective effect abolished when the autophagy gene Atg5 is knocked out [115].

PD-L1 overexpression is a well-known way to escape from recognition by the immune system, in particular in most aggressive tumors, such as TNBC. The recent and unexpected discovery linking PD-L1 trafficking directly to mitophagy invites new potential applications in immunotherapy. Small molecules or PROTACs designed to inhibit ATAD3A would “unlock” PINK1, forcing selective mitophagic degradation of PD-L1 and stripping tumor cells of their main immune-evasion shield. The clinical evidence behind this comes from the FUTURE trial, which highlighted that TNBC patients treated with anti-PD-1 plus nab-paclitaxel responded far better when their tumors showed PD-L1 localized at mitochondria rather than accumulated on the cell membrane [116].

Finally, restoring mitochondrial quality control in *BRCA*-deficient tumors could dampen the inflammatory signals that draw immunosuppressive cells into the tumor microenvironment.

## 10. Conclusions

Mitophagy has spent years in the shadow of its more famous cousin, autophagy, overlooked partly because it is harder to measure, and partly because the field kept looking for a single answer to what is, by nature, a moving target. What this review makes clear is that the question was never “does mitophagy help or hurt breast cancer,” but “which stage or molecular background.” That reframing is the real contribution here, and it is what turns mitophagy from an interesting mechanism into an actionable one.

## Figures and Tables

**Table 1:** Clinical and translational studies evaluating mitophagy-related markers in breast cancer.

Marker	Study Population and Clinical Context	Subtype/Stage	Method and Level of Analysis	Main Clinicopathological or Prognostic Finding	Interpretation and Limitation
PINK1 mRNA	Yaghoobi et al. (2020), [62] 54 paired breast tumor/adjacent-tissue samples	Breast cancer; *PINK1* showed no association with the different molecular subtypes	qRT-PCR; transcript level	PINK1 mRNA was reduced in tumor tissue and associated with mitotic rate.	Supports transcript-level downregulation, but provides no robust survival or treatment-response analysis.
PINK1 protein	Li et al. (2022) [63] 150 breast cancer tissues representing different molecular subtypes	Mixed molecular subtypes	Tissue microarray immunohistochemistry; protein level	Strong PINK1 staining was associated with a higher histological grade.	Supports a protein-level association with tumor aggressiveness; subtype-specific and multivariable prognostic analyses were limited.
PINK1 localization	Berthier et al. (2011), [64] normal and malignant breast tissue sections	Breast cancer versus normal tissue	Immunohistochemistry/immunofluorescence; subcellular localization	Tumor tissue showed diffuse cytoplasmic staining with membrane accumulation, whereas normal tissue showed a more punctate cytoplasmic pattern.	Suggests that localization may provide additional biological information; prognostic validation was not established.
PINK1 transcript	Li et al. (2022), [63] retrospective TCGA-BRCA cohort of 1070 cases	Pan-breast-cancer cohort; not resolved by molecular subtype	Public RNA-expression dataset; survival analysis	PINK1 was one of the six genes comprising a prognostic pyroptosis signature. Higher PINK1 expression was associated with shorter overall survival.	Retrospective transcriptomic association; lacks protein-level validation, subtype stratification, and direct flux measurement.
PINK1-related signature	Xiang et al. (2026), [93] transcriptomic analysis with retrospective immunohistochemical validation	TNBC	Bulk, single-cell and spatial transcriptomics plus IHC	High tumor-cell PINK1 expression was reported to be associated with recurrence and shorter disease-free survival.	Hypothesis-generating evidence; PINK1-specific prognostic independence and external validation required.
PRKN promoter methylation	Perego et al. (2024), [40] Paired breast cancer and adjacent normal tissue samples	Breast cancer; various subtypes	Methylation analysis/RT-qPCR	Promoter hypermethylation in breast cancer leads to PRKN downregulation. Demethylation restores expression and triggers an interferon/cGAS-STING immune response.	Demonstrates epigenetic suppression of mitophagy; provides a link to antitumor immunity, though direct clinical prognostic correlation requires larger prospective validation.
BNIP3	Chourasia et al. (2015), [46] Mouse models and clinical cohorts	TNBC progression/metastasis	Tissue IHC, transcriptomic analysis, and deletion mapping	Reduced BNIP3 expression or chromosome 10q26.3 deletion drives mitophagy defects, ROS accumulation, HIF-1α activation, and metastatic progression.	Highlights BNIP3 loss as a driver of aggressiveness and potential progression marker; clinical relevance remains complex due to divergent findings in node-positive vs. node-negative disease.
BNIP3	Koop et al. (2009), [48] 40 invasive breast cancer cases	Invasive breast carcinoma (ER+ and ER-)	Immunohistochemistry (IHC)	Nuclear localization correlates with cytoplasmic expression and with axillary lymph node status.	Suggests subcellular localization determines clinical impact; static IHC cannot distinguish active mitophagic flux from simple expression.
FUNDC1	Wu et al. (2019), [65] 102 breast cancer patient tissues and 44 normal controls	Advanced-stage breast cancer	Immunohistochemistry (IHC) and survival analysis	Strong cytoplasmic/nuclear-membrane staining in tumors; high expression correlates with larger tumor size, advanced stage, and reduced overall survival.	Supports an oncogenic role via Ca^2+^-NFATC1-BMI1 signaling; operates partly through non-mitophagic pathways, obscuring purely mitophagy-dependent effects.
MANF	Xiong et al. (2025), [58] 513 breast-cancer FFPE specimens under metabolic stress	Breast cancer (glucose-deprived contexts)	Expression analysis and functional survival assays	Elevated MANF expression correlates with poorer patient survival under nutrient stress.	Facilitates cell survival via PRKN-mediated mitophagy during starvation; functions as an adaptive pro-survival mechanism rather than a primary oncogene.
LC3B/HMGB1	Ladoire et al. (2015), [91] Patient cohorts receiving adjuvant/neoadjuvant chemotherapy	Breast cancer HER2+ and TNBC	IHC scoring of puncta in residual tumor tissue post-chemotherapy	Combined assessment of LC3B puncta and nuclear HMGB1 predicts residual risk of relapse.	Serves as a useful surrogate prognostic panel; reflects general autophagosome presence and immune infiltration rather than selective mitophagic flux.

## Data Availability

No new data were created or analyzed in this study. Data sharing is not applicable to this article.

## Abbreviations

The following abbreviations are used in this manuscript:

AAA	ATPases Associated with diverse cellular Activities
AKT	Protein Kinase B
AMPK	AMP-Activated Protein Kinase
ARIH1	Ariadne RBR E3 Ubiquitin Protein Ligase 1
ASCO	American Society of Clinical Oncology
ATAD3A	ATPase Family AAA Domain-Containing Protein 3A
ATG	Autophagy-Related Protein
ATG8	Autophagy-Related Protein 8
BC	Breast Cancer
BCSC	Breast Cancer Stem Cell
BCL2	B-cell Lymphoma 2
BMI1	BMI1 Proto-Oncogene, Polycomb Ring Finger Complex Component
BNIP3	BCL2 Interacting Protein 3
BNIP3L/NIX	BNIP3-Like Protein/NIP3-Like Protein X
BRCA1	Breast Cancer Gene 1
CAF	Cancer-Associated Fibroblast
CALCOCO2/NDP52	Calcium-Binding and Coiled-Coil Domain 2/Nuclear Dot Protein 52
Cdc42	Cell Division Control Protein 42
CDK6	Cyclin-Dependent Kinase 6
cGAS-STING	Cyclic GMP-AMP Synthase-Stimulator of Interferon Genes
CHMP2A/CHMP4B	Charged Multivesicular Body Protein 2A/4B
CHQ/HCQ	Chloroquine/Hydroxychloroquine
CSC	Cancer Stem Cell
DFS	Disease-Free Survival
DNA	Deoxyribonucleic Acid
DNM1L	Dynamin-1-Like Protein
DTC	Disseminated Tumor Cell
DTP	Drug-Tolerant Persister
EMT	Epithelial–Mesenchymal Transition
ER	Estrogen Receptor
ERK1/2	Extracellular Signal-Regulated Kinases 1/2
ESCRT-III	Endosomal Sorting Complex Required for Transport III
FAO	Fatty Acid Oxidation
FIS1	Mitochondrial Fission 1 Protein
FUNDC1	FUN14 Domain-Containing Protein 1
GABARAP	Gamma-Aminobutyric Acid Receptor-Associated Protein
GFP	Green Fluorescent Protein
GPCPD1	Glycerophosphocholine Phosphodiesterase1
HER2	Human Epidermal Growth Factor Receptor 2
HIF-1α	Hypoxia-Inducible Factor 1-Alpha
HMGB1	High Mobility Group Box 1
IGF-1	Insulin-Like Growth Factor 1
IHC	Immunohistochemistry
ITGB4	Integrin Subunit Beta 4
KO	Knock-Out
LC3/LC3B	Microtubule-Associated Protein 1A/1B-Light Chain 3/3B
LIR	LC3-Interacting Region
MANF	Mesencephalic Astrocyte-Derived Neurotrophic Factor
MAPK1/3	Mitogen-Activated Protein Kinase 1/3
MCT1	Monocarboxylate Transporter 1
MRPL52	Mitochondrial Ribosomal Protein L52
mRNA	Messenger Ribonucleic Acid
mt-Keima/mKeima	Mitochondrial-targeted Keima reporter
MUC1	Mucin 1
mTOR/mTORC1	Mechanistic Target of Rapamycin/Complex 1
NF-κB	Nuclear Factor Kappa-light-chain-enhancer of Activated B Cells
NFATC1	Nuclear Factor of Activated T-Cells 1
NIX	NIP3-Like Protein
NLRP3	NLR Family Pyrin Domain Containing 3
OMM	Outer Mitochondrial Membrane
OPTN	Optineurin
OS	Overall Survival
OXPHOS	Oxidative Phosphorylation
p53/TP53	Tumor Protein p53
PARL	Presenilin-Associated Rhomboid-Like Protein
PD-1	Programmed Cell Death Protein 1
PDK1	Pyruvate Dehydrogenase Kinase 1
PD-L1	Programmed Death-Ligand 1
PHB2	Prohibitin 2
PI3K	Phosphoinositide 3-Kinase
PINK1	PTEN-Induced Kinase 1
PLSCR3	Phospholipid Scramblase 3
PR	Progesterone Receptor
PRKN/Parkin	Parkin RBR E3 Ubiquitin Protein Ligase
PROTAC	Proteolysis Targeting Chimera
RCD	Regulated Cell Death
ROS	Reactive Oxygen Species
SQSTM1/p62	Sequestosome 1
TBK1	TANK-Binding Kinase 1
TGFβ1	Transforming Growth Factor Beta 1
TME	Tumor Microenvironment
TNBC	Triple-Negative Breast Cancer
TOM20	Translocase of Outer Mitochondrial Membrane 20
VPS4	Vacuolar Protein Sorting-Associated Protein 4
WIPI1	WD Repeat Domain, Phosphoinositide Interacting 1

## References

[1] Carvalho E., Canberk S., Schmitt F., Vale N. (2025). Molecular Subtypes and Mechanisms of Breast Cancer: Precision Medicine Approaches for Targeted Therapies. Cancers.

[2] Kannan K., Srinivasan A., Kannan A., Ali N. (2025). The Underlying Mechanisms and Emerging Strategies to Overcome Resistance in Breast Cancer. Cancers.

[3] Zhang Y., Ou Q., Hu C., Peng C., Li T., Huang X., Yang K., Li L., Li X., Xu Y. (2025). Dissecting regulated cell death states and transformation mechanisms in the evolutionary trajectory of 30 cancers. iScience.

[4] Wang M., Liu M., Yang C., Hu Y., Liao X., Liu Q. (2024). Autophagy Modulation in Therapeutic Strategy of Breast Cancer Drug Resistance. J. Cancer.

[5] Li A., Liu S., Xuan Y., Liang Y., Wang L., Zhao L. (2025). Distinct mechanisms of cell in breast cancer and their clinical implication. Cell Biol. Toxicol..

[6] Ashrafi G., Schwarz T.L. (2013). The pathways of mitophagy for quality control and clearance of mitochondria. Cell Death Differ..

[7] Wang Q., Sun Y., Li T.Y., Auwerx J. (2026). Mitophagy in the pathogenesis and management of disease. Cell Res..

[8] Narendra D.P., Jin S.M., Tanaka A., Suen D.-F., Gautier C.A., Shen J., Cookson M.R., Youle R.J. (2010). PINK1 is selectively stabilized on impaired mitochondria to activate Parkin. PLoS Biol..

[9] Hanna R.A., Quinsay M.N., Orogo A.M., Giang K., Rikka S., Gustafsson Å.B. (2012). Microtubule-associated protein 1 light chain 3 (LC3) interacts with Bnip3 protein to selectively remove endoplasmic reticulum and mitochondria via autophagy. J. Biol. Chem..

[10] Huang J., Pham V.T., Fu S., Huang G., Liu Y.G., Zheng L. (2025). Mitophagy’s impacts on cancer and neurodegenerative diseases: Implications for future therapies. J. Hematol. Oncol..

[11] Sandoval H., Thiagarajan P., Dasgupta S.K., Schumacher A., Prchal J.T., Chen M., Wang J. (2008). Essential role for Nix in autophagic maturation of erythroid cells. Nature.

[12] Liu L., Feng D., Chen G., Chen M., Zheng Q., Song P., Ma Q., Zhu C., Wang R., Qi W. (2012). Mitochondrial outer-membrane protein FUNDC1 mediates hypoxia-induced mitophagy in mammalian cells. Nat. Cell Biol..

[13] Sekine S., Youle R.J. (2018). PINK1 import regulation; a fine system to convey mitochondrial stress to the cytosol. BMC Biol..

[14] Okatsu K., Koyano F., Kimura M., Kosako H., Saeki Y., Tanaka K., Matsuda N. (2015). Phosphorylated ubiquitin chain is the genuine Parkin receptor. J. Cell Biol..

[15] Lagunas-Rangel F.A. (2025). Sirtuins in mitophagy: Key gatekeepers of mitochondrial quality. Mol. Cell. Biochem..

[16] Heo J.M., Ordureau A., Paulo J.A., Rinehart J., Harper J.W. (2015). The PINK1-PARKIN Mitochondrial Ubiquitylation Pathway Drives a Program of OPTN/NDP52 Recruitment and TBK1 Activation to Promote Mitophagy. Mol. Cell.

[17] Herhaus L. (2021). TBK1 (TANK-binding kinase 1)-mediated regulation of autophagy in health and disease-NC-ND license. Matrix Biol..

[18] Richter B., Sliter D.A., Herhaus L., Stolz A., Wang C., Beli P., Zaffagnini G., Wild P., Martens S., Wagner S.A. (2016). Phosphorylation of OPTN by TBK1 enhances its binding to Ub chains and promotes selective autophagy of damaged mitochondria. Proc. Natl. Acad. Sci. USA.

[19] Nguyen T.N., Sawa-Makarska J., Khuu G., Lam W.K., Adriaenssens E., Fracchiolla D., Shoebridge S., Bernklau D., Padman B.S., Skulsuppaisarn M. (2023). Unconventional initiation of PINK1/Parkin mitophagy by Optineurin. Mol. Cell.

[20] Li S., Zhang J., Liu C., Wang Q., Yan J., Hui L., Jia Q., Shan H., Tao L., Zhang M. (2021). The Role of Mitophagy in Regulating Cell Death. Oxidative Med. Cell. Longev..

[21] Adriaenssens E., Schaar S., Cook A.S.I., Stuke J.F.M., Sawa-Makarska J., Nguyen T.N., Ren X., Schuschnig M., Romanov J., Khuu G. (2025). Reconstitution of BNIP3/NIX-mitophagy initiation reveals hierarchical flexibility of the autophagy machinery. Nat. Cell Biol..

[22] Tang C., Dong Z. (2024). Ubiquitin-independent mitophagy: Mechanisms and pathophysiological functions. Mitophagy in Health and Disease: Mechanisms, Health Implications, and Therapeutic Opportunities.

[23] Yan J., Chen X., Choksi S., Liu Z.G. (2025). TGFB signaling induces mitophagy via PLSCR3-mediated cardiolipin externalization in conjunction with a BNIP3L/NIX-, BNIP3-, and FUNDC1-dependent mechanism. Autophagy.

[24] García-Chávez D., Domínguez-Martín E., Kawasaki L., Ongay-Larios L., Ruelas-Ramírez H., Mendoza-Martinez A.E., Pardo J.P., Funes S., Coria R. (2024). Prohibitins, Phb1 and Phb2, function as Atg8 receptors to support yeast mitophagy and also play a negative regulatory role in Atg32 processing. Autophagy.

[25] Zhen Y., Spangenberg H., Munson M.J., Brech A., Schink K.O., Tan K.-W., Sørensen V., Wenzel E.M., Radulovic M., Engedal N. (2020). ESCRT-mediated phagophore sealing during mitophagy. Autophagy.

[26] Vara-Perez M., Felipe-Abrio B., Agostinis P. (2019). Mitophagy in cancer: A tale of adaptation. Cells.

[27] Kongara S., Karantza V. (2012). The interplay between autophagy and ROS in tumorigenesis. Front. Oncol..

[28] Tan X., Na J., Tian X., Zhu R., Su Y., Chen Y., Zhong L. (2026). Mitophagy-driven multidimensional regulation of tumor immune evasion and context-dependent therapeutic strategies. J. Transl. Med..

[29] Chen S., Li Q., Shi H., Li F., Duan Y., Guo Q. (2024). New insights into the role of mitochondrial dynamics in oxidative stress-induced diseases. Biomed. Pharmacother..

[30] K D., Shee M., Roy P.K., Mandal M. (2026). Reactive oxygen species in cell signaling and cell death: A redox perspective on metabolic and inflammatory disorders. Cell Death.

[31] Kowalczyk P., Sulejczak D., Kleczkowska P., Bukowska-Ośko I., Kucia M., Popiel M., Wietrak E., Kramkowski K., Wrzosek K., Kaczyńska K. (2021). Mitochondrial oxidative stress—A causative factor and therapeutic target in many diseases. Int. J. Mol. Sci..

[32] Lin J., Chen X., Du Y., Li J., Guo T., Luo S. (2024). Mitophagy in Cell Death Regulation: Insights into Mechanisms and Disease Implications. Biomolecules.

[33] Lee S.B., Kim J.J., Nam H.-J., Gao B., Yin P., Qin B., Yi S.-Y., Ham H., Evans D., Kim S.-H. (2015). Parkin Regulates Mitosis and Genomic Stability through Cdc20/Cdh1. Mol. Cell.

[34] Fujiwara M., Marusawa H., Wang H.-Q., Iwai A., Ikeuchi K., Imai Y., Kataoka A., Nukina N., Takahashi R., Chiba T. (2008). Parkin as a tumor suppressor gene for hepatocellular carcinoma. Oncogene.

[35] Cesari R., Martin E.S., Calin G.A., Pentimalli F., Bichi R., McAdams H., Trapasso F., Drusco A., Shimizu M., Masciullo V. (2003). Parkin, a gene implicated in autosomal recessive juvenile parkinsonism, is a candidate tumor suppressor gene on chromosome 6q25–q27. Proc. Natl. Acad. Sci. USA.

[36] Tay S.P., Yeo C.W., Chai C., Chua P.-J., Tan H.-M., Ang A.X., Yip D.L., Sung J.-X., Tan P.H., Bay B.-H. (2010). Parkin enhances the expression of cyclin-dependent kinase 6 and negatively regulates the proliferation of breast cancer cells. J. Biol. Chem..

[37] Gupta A., Anjomani-Virmouni S., Koundouros N., Poulogiannis G. (2017). PARK2 loss promotes cancer progression via redox-mediated inactivation of PTEN. Mol. Cell. Oncol..

[38] Liu J., Zhang C., Zhao Y., Yue X., Wu H., Huang S., Chen J., Tomsky K., Xie H., Khella C.A. (2017). Parkin targets HIF-1α for ubiquitination and degradation to inhibit breast tumor progression. Nat. Commun..

[39] Kim H., Ronai Z.A. (2024). Parkin paves the path to antitumor immunity: Expanding Parkin’s role as a tumor suppressor. J. Clin. Investig..

[40] Perego M., Yeon M., Agarwal E., Milcarek A.T., Bertolini I., Camisaschi C., Ghosh J.C., Tang H.-Y., Grandvaux N., Ruscetti M. (2024). Parkin activates innate immunity and promotes antitumor immune responses. J. Clin. Investig..

[41] Chen H., Li Y., Chen Z., Xie L., Li W., Zhu Y., Lu D., Hong X., Koeffler H.P., Wu W. (2020). Abstract 72: PARK2 enhances chemosensitivity of antimicrotubule drugs in breast cancer via promoting degradation of BCL-2. Cancer Res..

[42] Zhang C., Lin M., Wu R., Wang X., Yang B., Levine A.J., Hu W., Feng Z. (2011). Parkin, a p53 target gene, mediates the role of p53 in glucose metabolism and the Warburg effect. Proc. Natl. Acad. Sci. USA.

[43] Zheng R., Yao Q., Xie G., Du S., Ren C., Wang Y., Yuan Y. (2015). TAT-ODD-p53 Enhances the Radiosensitivity of Hypoxic Breast Cancer Cells by Inhibiting Parkin-Mediated Mitophagy. Oncotarget.

[44] Yin K., Lee J., Liu Z., Kim H., Martin D.R., Wu D., Liu M., Xue X. (2021). Mitophagy protein PINK1 suppresses colon tumor growth by metabolic reprogramming via p53 activation and reducing acetyl-CoA production. Cell Death Differ..

[45] Guo Z.J., Yu Q., Sha R., Li W., Dai H.J. (2026). PINK1 mediated mitophagy enhances breast cancer proliferation through metabolic reprogramming. Oncol. Rep..

[46] Chourasia A.H., Tracy K., Frankenberger C., Boland M.L., Sharifi M.N., Drake L.E., Sachleben J.R., Asara J.M., Locasale J.W., Karczmar G.S. (2015). Mitophagy defects arising from BNip3 loss promote mammary tumor progression to metastasis. EMBO Rep..

[47] Vykoukal J., Chen Y., Zuo M., Ballarò R., Hong M.J., Krishna H., Rodriquez-Perera D.B., Katayama H., Irajizad E., Wu R. (2025). Vesicle-mediated mitochondrial clearance presents an actionable metabolic vulnerability in triple-negative breast cancer. Cell Rep. Med..

[48] Koop E.A., van Laar T., van Wichen D.F., de Weger R.A., Wall E., van Diest P.J. (2009). Expression of BNIP3 in invasive breast cancer: Correlations with the hypoxic response and clinicopathological features. BMC Cancer.

[49] Tan E.Y., Campo L., Han C., Turley H., Pezzella F., Gatter K.C., Harris A.L., Fox S.B. (2007). BNIP3 as a progression marker in primary human breast cancer opposing functions in in situ versus invasive cancer. Clin. Cancer Res..

[50] Chen C., Xiang A., Lin X., Guo J., Liu J., Hu S., Rui T., Ye Q. (2024). Mitophagy: Insights into its signaling molecules, biological functions, and therapeutic potential in breast cancer. Cell Death Discov..

[51] Chen Q., Lei J.H., Bao J., Wang H., Hao W., Li L., Peng C., Masuda T., Miao K., Xu J. (2020). BRCA1 Deficiency Impairs Mitophagy and Promotes Inflammasome Activation and Mammary Tumor Metastasis. Adv. Sci..

[52] Deng R., Zhang H.-L., Huang J.-H., Cai R.-Z., Wang Y., Chen Y.-H., Hu B.-X., Ye Z.-P., Li Z.-L., Mai J. (2021). MAPK1/3 kinase-dependent ULK1 degradation attenuates mitophagy and promotes breast cancer bone metastasis. Autophagy.

[53] Wang S., Long H., Hou L., Feng B., Ma Z., Wu Y., Zeng Y., Cai J., Zhang D.-W., Zhao G. (2023). The mitophagy pathway and its implications in human diseases. Signal Transduct. Target. Ther..

[54] Carretero-Fernández M., Cabrera-Serrano A.J., Sánchez-Maldonado J.M., Ruiz-Durán L., Jiménez-Romera F., García-Verdejo F.J., González-Olmedo C., Cardús A., Díaz-Beltrán L., Gutiérrez-Bautista J.F. (2025). Autophagy and oxidative stress in solid tumors: Mechanisms and therapeutic opportunities. Crit. Rev. Oncol./Hematol..

[55] Li H., Bai X., Yang T., Wu C., Liu Y., Shan C., Lin R., Wen X., Cheng A. (2025). HIF-1α Activates Mitophagy through BNIP3 to Inhibit IPEC-J2 Apoptosis under Heat Stress. J. Agric. Food Chem..

[56] Liu Y., Zhang H., Liu Y., Zhang S., Su P., Wang L., Li Y., Liang Y., Wang X., Zhao W. (2023). Hypoxia-induced GPCPD1 depalmitoylation triggers mitophagy via regulating PRKN-mediated ubiquitination of VDAC1. Autophagy.

[57] Li X., Wang M., Li S., Chen Y., Wang M., Wu Z., Sun X., Yao L., Dong H., Song Y. (2021). HIF-1-induced mitochondrial ribosome protein L52: A mechanism for breast cancer cellular adaptation and metastatic initiation in response to hypoxia. Theranostics.

[58] Xiong Z., Yang L., Zhang C., Huang W., Zhong W., Yi J., Feng J., Zouxu X., Song L., Wang X. (2025). MANF facilitates breast cancer cell survival under glucose-starvation conditions via PRKN-mediated mitophagy regulation. Autophagy.

[59] Guido C., Whitaker-Menezes D., Capparelli C., Balliet R., Lin Z., Pestell R.G., Howell A., Aquila S., Andò S., Martinez-Outschoorn U. (2012). Metabolic reprogramming of cancer-associated fibroblasts by TGF-β drives tumor growth: Connecting TGF-β signaling with ‘Warburg-like’ cancer metabolism and L-lactate production. Cell Cycle.

[60] Kim T.H., Lim S.H., Lee H., Chae Y.C., Min D.S. (2026). Metabolic crosstalk among cancer-associated fibroblasts, adipocytes and immune cells as an immunosuppressive tumor microenvironment driver. Exp. Mol. Med..

[61] Ma C., Teoh H.K., Zhao Y., Wang Y., Zhao J., Liu Y., Zhao C., Ong H.T. (2026). Reprogramming the immunosuppressive breast cancer microenvironment: Integrating cellular, metabolic, and stromal targets for rational immunotherapy. Front. Immunol..

[62] Yaghoobi H., Azizi H., Oskooei V.K., Taheri M., Ghafouri-Fard S. (2020). PTEN-induced putative kinase 1 (PINK1) down-regulation in breast cancer samples in association with mitotic rate. Meta Gene.

[63] Li J., Xu X., Huang H., Li L., Chen J., Ding Y., Ping J. (2022). Pink1 promotes cell proliferation and affects glycolysis in breast cancer. Exp. Biol. Med..

[64] Berthier A., Navarro S., Jiménez-Sáinz J., Roglá I., Ripoll F., Cervera J., Pulido R. (2011). PINK1 displays tissue-specific subcellular location and regulates apoptosis and cell growth in breast cancer cells. Hum. Pathol..

[65] Wu L., Zhang D., Zhou L., Pei Y., Zhuang Y., Cui W., Chen J. (2019). FUN14 domain-containing 1 promotes breast cancer proliferation and migration by activating calcium-NFATC1-BMI1 axis. EBioMedicine.

[66] Hui L., Wu H., Wang T.-W., Yang N., Guo X., Jang X.-J. (2019). Hydrogen peroxide-induced mitophagy contributes to laryngeal cancer cells survival via the upregulation of FUNDC1. Clin. Transl. Oncol..

[67] Rui K., Qiu J., Li M., Huang J., Wang T., Yin K. (2026). Crosstalk between mitophagy and breast cancer: Mechanisms of action and clinical applications. J. Cancer Res. Clin. Oncol..

[68] Yiu L., Chong Y.W., Yan S., Tsang S.Y. (2026). Role of mitophagy in breast cancer: Mitophagy-apoptosis balance and reactive oxygen species play determining role. Front. Physiol..

[69] Li Q., Chu Y., Li S., Yu L., Deng H., Liao C., Liao X., Yang C., Qi M., Cheng J. (2022). The oncoprotein MUC1 facilitates breast cancer progression by promoting Pink1-dependent mitophagy via ATAD3A destabilization. Cell Death Dis..

[70] Liu D., Li X., Zeng B., Zhao Q., Chen H., Zhang Y., Chen Y., Wang J., Xing H.R. (2023). Exosomal microRNA-4535 of Melanoma Stem Cells Promotes Metastasis by Inhibiting Autophagy Pathway. Stem Cell Rev. Rep..

[71] Lu J., Zheng X., Li F., Yu Y., Chen Z., Liu Z., Wang Z., Xu H., Yang W. (2017). Tunneling Nanotubes Promote Intercellular Mitochondria Transfer Followed by Increased Invasiveness in Bladder Cancer Cells. Oncotarget.

[72] Caicedo A., Fritz V., Brondello J.-M., Ayala M., Dennemont I., Abdellaoui N., de Fraipont F., Moisan A., Prouteau C.A., Boukhaddaoui H. (2015). MitoCeption as a new tool to assess the effects of mesenchymal stem/stromal cell mitochondria on cancer cell metabolism and function. Sci. Rep..

[73] Xie X.Q., Yang Y., Wang Q., Liu H.-F., Fang X.-Y., Li C.-L., Jiang Y.-Z., Wang S., Zhao H.-Y., Miao J.-Y. (2023). Targeting ATAD3A-PINK1-mitophagy axis overcomes chemoimmunotherapy resistance by redirecting PD-L1 to mitochondria. Cell Res..

[74] Romaniuk-Drapała A., Totoń E., Taube M., Idzik M., Rubiś B., Lisiak N. (2024). Breast Cancer Stem Cells and Tumor Heterogeneity: Characteristics and Therapeutic Strategies. Cancers.

[75] Smith A.G., Macleod K.F. (2019). Autophagy, cancer stem cells and drug resistance. J. Pathol..

[76] Mauro-Lizcano M., Sotgia F., Lisanti M.P. (2024). Mitophagy and cancer: Role of BNIP3/BNIP3L as energetic drivers of stemness features, ATP production, proliferation, and cell migration. Aging.

[77] Li Y., Chen H., Lu D., Koeffler H.P., Zhang Y., Yin D. (2023). Mitophagy is a novel protective mechanism for drug-tolerant persister (DTP) cancer cells. Autophagy.

[78] Zhang Z., Zhao Q., Xu Q., Deng Q., Hua A., Wang X., Yang X., Li Z. (2025). A mitochondria-interfering nanocomplex cooperates with photodynamic therapy to boost antitumor immunity. Biomaterials.

[79] Howley B.V., Mohanty B., Dalton A., Grelet S., Karam J., Dincman T., Howe P.H. (2022). The ubiquitin E3 ligase ARIH1 regulates hnRNP E1 protein stability, EMT and breast cancer progression. Oncogene.

[80] Liu X., Cen X., Wu R., Chen Z., Xie Y., Wang F., Shan B., Zeng L., Zhou J., Xie B. (2023). ARIH1 activates STING-mediated T-cell activation and sensitizes tumors to immune checkpoint blockade. Nat. Commun..

[81] Cocco S., Leone A., Piezzo M., Caputo R., Di Lauro V., Di Rella F., Fusco G., Capozzi M., di Gioia G., Budillon A. (2020). Targeting autophagy in breast cancer. Int. J. Mol. Sci..

[82] Ahmadpour S.T., Desquiret-Dumas V., Yikilmaz U., Dartier J., Domingo I., Wetterwald C., Orre C., Gueguen N., Brisson L., Mahéo K. (2021). Doxorubicin-induced autophagolysosome formation is partly prevented by mitochondrial ros elimination in dox-resistant breast cancer cells. Int. J. Mol. Sci..

[83] Zhou J., Li G., Zheng Y., Shen H.-M., Hu X., Ming Q.-L., Huang C., Li P., Gao N. (2015). A novel autophagy/mitophagy inhibitor liensinine sensitizes breast cancer cells to chemotherapy through DNM1L-mediated mitochondrial fission. Autophagy.

[84] Naso F.D., Bruqi K., Manzini V., Chiurchiù V., D’onofrio M., Arisi I., Strappazzon F. (2024). miR-218-5p and doxorubicin combination enhances anticancer activity in breast cancer cells through Parkin-dependent mitophagy inhibition. Cell Death Discov..

[85] Pu Y., Xu F., He A., Li R., Wang X., Zhou L., Sun H., Zhang Y., Xia Y. (2025). Repurposing chlorpromazine for the treatment of triple-negative breast cancer growth and metastasis based on modulation of mitochondria-mediated apoptosis and autophagy/mitophagy. Br. J. Cancer.

[86] Ye Y., Zeng Q., Ou Z., Ju X., Liao Q., Li C., Zhang D., Wei Y., Zhang X., Wu K. (2026). Mitochondrial pyruvate dehydrogenase kinase 1 drives bevacizumab resistance and malignant phenotype of TNBC by enhancing mitophagy. Pharmacol. Res..

[87] Assi M., Kimmelman A.C. (2023). Impact of context-dependent autophagy states on tumor progression. Nat. Cancer.

[88] Lefort S., Joffre C., Kieffer Y., Givel A.-M., Bourachot B., Zago G., Bieche I., Dubois T., Meseure D., Vincent-Salomon A. (2014). Inhibition of autophagy as a new means of improving chemotherapy efficiency in high-LC3B triple-negative breast cancers. Autophagy.

[89] Klionsky D.J., Abdel-Aziz A.K., Abdelfatah S., Abdellatif M., Abdoli A., Abel S., Abeliovich H., Abildgaard M.H., Abudu Y.P., Acevedo-Arozena A. (2021). Guidelines for the use and interpretation of assays for monitoring autophagy (4th edition). Autophagy.

[90] Zhao H., Zhang W., Du Y., Kuang Z., Tan Y., Chen Y., Ma Y., Zou F. (2026). Comprehensive analysis of mitophagy-related genes reveals prognostic signatures in breast cancer: Based on immune landscapes and treatment target predict. Front. Immunol..

[91] Ladoire S., Penault-Llorca F., Senovilla L., Dalban C., Enot D., Locher C., Prada N., Poirier-Colame V., Chaba K., Arnould L. (2015). Combined evaluation of LC3B puncta and HMGB1 expression predicts residual risk of relapse after adjuvant chemotherapy in breast cancer. Autophagy.

[92] Petry I.B., Fieber E., Schmidt M., Gehrmann M., Gebhard S., Hermes M., Schormann W., Selinski S., Freis E., Schwender H. (2010). ERBB2 induces an antiapoptotic expression pattern of Bcl-2 family members in node-negative breast cancer. Clin. Cancer Res..

[93] Xiang L., Rao J., Ma A., Zhang Y., Li C., Xie T., Xue H., Chen Z., Liu T., Yuan J. (2026). Integrating bulk, single-cell, and spatial transcriptomics to identify a novel pyroptosis-related gene signature for predicting prognosis and tumor immune landscape in triple-negative breast cancer. Front. Immunol..

[94] Liu G., Yu G., Yin D., Ma J. (2025). Discovery of a new mitophagy-related gene signature for predicting the outlook and immunotherapy in triple-negative breast cancer. Sci. Rep..

[95] Lin H.Y., Wu H.J., Chu P.Y. (2023). Multi-omics and experimental analysis unveil theragnostic value and immunological roles of inner membrane mitochondrial protein (IMMT) in breast cancer. J. Transl. Med..

[96] Wang Z., Zhao Y., Zhang L. (2024). Emerging trends and hot topics in the application of multi-omics in drug discovery: A bibliometric and visualized study. Curr. Pharm. Anal..

[97] Katayama H., Kogure T., Mizushima N., Yoshimori T., Miyawaki A. (2011). A sensitive and quantitative technique for detecting autophagic events based on lysosomal delivery. Chem. Biol..

[98] McWilliams T.G., Prescott A.R., Allen G.F., Tamjar J., Munson M.J., Thomson C., Muqit M.M., Ganley I.G. (2016). Mito-QC illuminates mitophagy and mitochondrial architecture in vivo. J. Cell Biol..

[99] Jiménez-Loygorri J.I., Jiménez-García C., Viedma-Poyatos Á., Boya P. (2024). Fast and quantitative mitophagy assessment by flow cytometry using the mito-QC reporter. Front. Cell Dev. Biol..

[100] Montava-Garriga L., Ganley I.G. (2020). Outstanding Questions in Mitophagy: What We Do and Do Not Know. J. Mol. Biol..

[101] Uoselis L., Nguyen T.N., Lazarou M. (2023). Mitochondrial degradation: Mitophagy and beyond. Mol. Cell.

[102] Liu Y.T., Sliter D.A., Shammas M.K., Huang X., Wang C., Calvelli H., Maric D.S., Narendra D.P. (2021). Mt-Keima detects PINK1-PRKN mitophagy in vivo with greater sensitivity than mito-QC. Autophagy.

[103] Xiao B., Deng X., Zhou W., Tan E.K. (2016). Flow cytometry-based assessment of mitophagy using mitotracker. Front. Cell. Neurosci..

[104] Sugiura A., McLelland G., Fon E.A., McBride H.M. (2014). A new pathway for mitochondrial quality control: Mitochondrial-derived vesicles. EMBO J..

[105] De Sanctis J.B., Charris J., Blanco Z., Ramírez H., Martínez G.P., Mijares M.R. (2022). Molecular Mechanisms of Chloroquine and Hydroxychloroquine Used in Cancer Therapy. Anticancer Agents Med. Chem..

[106] Cocco S., Leone A., Roca M.S., Lombardi R., Piezzo M., Caputo R., Ciardiello C., Costantini S., Bruzzese F., Sisalli M.J. (2022). Inhibition of autophagy by chloroquine prevents resistance to PI3K/AKT inhibitors and potentiates their antitumor effect in combination with paclitaxel in triple negative breast cancer models. J. Transl. Med..

[107] DeMichele A., Clark A.S., Shea E., Bayne L.J., Sterner C.J., Rohn K., Dwyer S., Pan T.-C., Nivar I., Chen Y. (2025). Targeting dormant tumor cells to prevent recurrent breast cancer: A randomized phase 2 trial. Nat. Med..

[108] Raghavendra A.S., Kettner N.M., Kwiatkowski D., Damodaran S., Wang Y., Ramirez D., Gombos D.S., Hunt K.K., Shen Y., Keyomarsi K. (2025). Phase I trial of hydroxychloroquine to enhance palbociclib and letrozole efficacy in ER+/HER2− breast cancer. npj Breast Cancer.

[109] Bardia A., Santa-Maria C.A., Jacobs L.K., Cimino-Mathews A., Huang P., Russell S., Camp M., Habibi M., Lange J.R., Jeter S. (2013). Digoxin as an inhibitor of global hypoxia inducible factor-1α (HIF1α) expression and downstream targets in breast cancer: Dig-HIF1 pharmacodynamic trial. J. Clin. Oncol..

[110] McDonald P.C., Chia S., Bedard P.L.M., Chu Q., Lyle M., Tang L., Singh M., Zhang Z., Supuran C.T., Renouf D.J. (2020). A Phase 1 Study of SLC-0111, a Novel Inhibitor of Carbonic Anhydrase IX, in Patients With Advanced Solid Tumors. Am. J. Clin. Oncol..

[111] Halford S., Veal G.J., Wedge S.R., Payne G.S., Bacon C.M., Sloan P., Dragoni I., Heinzmann K., Potter S., Salisbury B.M. (2023). A Phase I Dose-Escalation Study of AZD3965, an Oral Monocarboxylate Transporter 1 Inhibitor, in Patients with Advanced Cancer. Clin. Cancer Res..

[112] Koundouros N., Poulogiannis G. (2018). Phosphoinositide 3-Kinase/Akt signaling and redox metabolism in cancer. Front. Oncol..

[113] Singh A., D’aMico D., Andreux P.A., Fouassier A.M., Blanco-Bose W., Evans M., Aebischer P., Auwerx J., Rinsch C. (2022). Urolithin A improves muscle strength, exercise performance, and biomarkers of mitochondrial health in a randomized trial in middle-aged adults. Cell Rep. Med..

[114] Denk D., Singh A., Kasler H., Boquet L.A., D’Amico D., Gorol J., Greten F. (2024). Impact of urolithin A supplementation, a mitophagy activator on mitochondrial health of immune cells (MitoIMMUNE): A randomized, double-blind, placebo-controlled trial in healthy adults. J. Clin. Oncol..

[115] Tong D., Hill J.A. (2017). Spermidine Promotes Cardioprotective Autophagy. Circ. Res..

[116] Jiang Y.Z., Liu Y., Xiao Y., Hu X., Jiang L., Zuo W.-J., Ma D., Ding J., Zhu X., Zou J. (2021). Molecular subtyping and genomic profiling expand precision medicine in refractory metastatic triple-negative breast cancer: The FUTURE trial. Cell Res..

